# Visible Multiphoton Dissociation of Chromophore-Tagged Peptides

**DOI:** 10.1007/s13361-017-1733-9

**Published:** 2017-07-28

**Authors:** Mathilde Bouakil, Alexander Kulesza, Steven Daly, Luke MacAleese, Rodolphe Antoine, Philippe Dugourd

**Affiliations:** Université Lyon, Université Claude Bernard Lyon 1, CNRS, Institut Lumière Matière, F-69622 Villeurbanne, France

**Keywords:** Fragmentation, Photodissociation, Peptides, Visible, Collision induced dissociation, Multiphoton

## Abstract

The visible photodissociation mechanisms of QSY7-tagged peptides of increasing size have been investigated by coupling a mass spectrometer and an optical parametric oscillator laser beam. The experiments herein consist of energy resolved collision- and laser-induced dissociation measurements on the chromophore-tagged peptides. The results show that fragmentation occurs by similar channels in both activation methods, but that the branching ratios are vastly different. Observation of a size-dependent minimum laser pulse energy required to induce fragmentation, and collisional cooling rates in time resolved experiments show that laser-induced dissociation occurs through the absorption of multiple photons by the chromophore and the subsequent heating through vibrational energy redistribution. The differences in branching ratio between collision- and laser-induced dissociation can then be understood by the highly anisotropic energy distribution following absorption of a photon.

**Electronic supplementary material:**

The online version of this article (doi:10.1007/s13361-017-1733-9) contains supplementary material, which is available to authorized users.

**Graphical Abstract:**
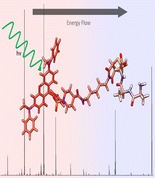
ᅟ

## Introduction

There are multitudes of activation mechanisms that can be used to identify and study a diverse range of properties in molecules. These include collision-based methods such as collision-induced dissociation (CID) and electron impact, absorption-based methods such as UV photodissociation (UVPD) and infrared multiphoton dissociation (IRMPD), and electron-based techniques like electron capture/transfer dissociation (ECD/ETD) [1–8]. CID, for example, is known to produce extensive *b/y* fragmentation in peptides, and can be used in order to sequence unknown proteins [1]. By contrast, ECD/ETD produce predominantly *c/z* ions, and more importantly fragmentation occurs at different points along the peptide backbone, thus giving different sequence coverage [7].

Of the techniques that rely on photon absorption, IRMPD and UVPD are by far the most common [4–6, 9]. The nature of the fragmentation observed depends upon the energy of each photon and the timescale for the deposition of the energy required to induce fragmentation. In IRMPD, an IR photon (typically of wavelength 10.6 μm) is absorbed by the ion to produce a vibrationally excited ion, which may undergo further absorption events. When enough photons have been absorbed for the internal energy of the ion to cross the dissociation threshold, fragmentation can ensue. Depending upon the rate of excitation, there are several possible pathways by which IRMPD may proceed. If the photon flux is low enough that intramolecular vibrational energy redistribution (IVR) can take place, the energy of each absorbed photon will be distributed throughout the ion, and additional photons will cause a gradual heating of the system until the system dissociates. Activation timescales can be as high as several seconds [10, 11]. Deactivation by IR photon emission and/or collisions occur on similar timescales, which may significantly reduce the heating rate of the system. Contrastingly, if the photon flux is high, or there is a strong IR chromophore, multiple excitations of a single normal mode may be possible before IVR can take place, and this leads to an asymmetric heating of the ion. Here, activation timescales can be much shorter, of the order of microseconds or less [10, 12]. Nevertheless, both these examples require multiple activation events over a time period long compared with the unimolecular reaction rate and may be classed as slow activation mechanisms.

In contrast to IRMPD, the photon energy is much higher in UVPD and photodissociation may occur after the absorption of a single photon. For example, 192 nm photons are used to photoexcite the peptide bond and cause extensive fragmentation in peptides and proteins [5]. Here, the timescale for the activation event is the timescale of photoexcitation, ~10^–15^ s, which is much faster than the unimolecular reaction rate and therefore can be classed as a fast activation method. Indeed, at high photon energy, fast dissociation may occur before equilibrium is reached, leading to differences in the fragments observed in UVPD versus IRMPD or CID.

Absorption of visible light is, energetically speaking, bounded by the limiting cases of IRMPD and UVPD, and the photon energy is approximately the same as many bond dissociation energies. Absorption of visible light is uncommon as an activation mechanism, mainly because the majority of biomolecules do not possess a natural visible chromophore. There are certain classes of molecules, for example the radical cations of polycyclic aromatic hydrocarbons (PAHs), which absorb in the visible region of the spectrum. Joblin et al. showed that photofragmentation of various PAHs occurs via a sequential multiphoton process [13]. However, in most cases where visible light has been used, a synthetic chromophore group must be tagged to the biomolecule being studied. Fluorescent tags have also been used as sensitive probes of the structure of biomolecules, in particular in FRET-based experiments [14–18]; Hahn and Grotemeyer used rhodamine 101 as a tag to induce photofragmentation in a series of oligosaccharides [19]. Fluorescence quenchers have also been used to probe biomolecules, both as tags for visible photodissociation (VisPD) quantification approaches by selected reaction monitoring (photo-SRM) and as reporters of FRET [20–25]. As fluorescence is not available as a relaxation mechanism in quenchers, upon absorption of a photon there will be a competition between unimolecular decay and redistribution of energy from the chromophore to the tagged peptide or protein. Visible absorption therefore represents an intermediate case to IRMPD and UVPD in terms of photophysical processes that occur. The activation can be fast; it is possible to induce VisPD with a single nanosecond laser pulse where the maximum activation time will be several nanoseconds. However, depending upon bond dissociation energy values and how the competition between unimolecular decay and IVR unfolds, it may be possible that several photons are required to induce fragmentation, in common with IRMPD. This combination of fast activation with the potential for multiple absorption events provides VisPD with a unique activation method.

In this paper, we explore the VisPD dynamics of the rhodamine derivative QSY7 alone and tagged onto a series of peptides of increasing size. It is known that QSY7 relaxes by undergoing internal conversion (IC), and possesses a gas-phase absorption maximum of 545 nm [23]. These systems will therefore allow probing how photofragmentation—which occurs via IC—proceeds, and how the size of the system influences these dynamics. Furthermore, the use of visible photons allows a detailed exploration of the photophysics of chromophore-tagged peptides in the visible region. The insight into the degree of energy dispersion in these large molecules after initial energy deposition by photons gets at questions that have long been both interesting and difficult in the study of unimolecular dissociation. It also has analytical implications in techniques rapidly developing for MS^2^ measurements, including the use of near-UV and visible chromophores [26, 27]. Action spectroscopy to investigate structure and reactivity of large gas-phase ions is also emerging as a new technique in structural biology strongly related to native MS.

## Materials and Methods

QSY7 C_5_ maleimide (Q, Molecular Probes, Eugene, OR, USA) was dissolved to a concentration of 1 mg/100 μL in DMSO. Ace-Ala_n_Cys-NH_2_ with n = 1, 2, 4, 6 and 8, respectively (abbreviated to AC, A_2_C, A_4_C, A_6_C, and A_8_C in the rest of the manuscript) were purchased from Genecust, Ellange, Luxembourg (Figure S1). AC, A_2_C, and A_4_C were dissolved in H_2_O to a concentration of 1 mg/mL, A_6_C and A_8_C were dissolved in trifluoroethanol at 1 mg/mL. Chromophore tagging was performed in 1:1 H_2_O:CH_3_OH at a final concentration of 10 μM by adding an equimolar quantity of chromophore and each peptide stock solution independently. The chromophores grafting is spontaneous in these conditions (click chemistry). For use in the electrospray ionization source, equal volumes of the reaction solutions were combined and injected directly into the mass spectrometer.

### Mass Spectrometry and Optical Spectroscopy

All mass spectrometry was performed on a modified dual linear ion trap (LTQ Velos, Thermo Fisher Scientific, San Jose, CA, USA) [28]. Ions were generated using an electrospray ionization (ESI) source. Ions can be mass selected, stored, and activated in either a high or low pressure ion trap. The mass spectrometer is modified by adapting a 1 in. diameter fused silica window on the vacuum manifold, on axis with the ion traps. Each trapping electrode has a 1–2 mm hole that allows coupling of the trapped ions and laser in either trap. In order to optimize laser transmission, the hole in the electrode closest to the fused silica window is enlarged to 5 mm. CID experiments are performed in the high-pressure trap. Each ion is mass selected and activated at normalized collision energies (NCE) between 0 and 40, and mass spectra are averaged over 2 min [29]. Branching ratios are calculated as *I*_*f*_/*I*_*tot*_ where *I*_*f*_ is the intensity of fragment *f* and *I*_*tot*_ is the total intensity. The fragmentation yield is calculated as $- \log \left(\frac{I_p}{I_{tot}}\right),$ where *I*_*p*_ is the intensity of the parent peak.

The lasers used are a Horizon OPO and PantherEx OPO (Continuum, Santa Clara, CA, USA), both of which have repetition rates of 10 Hz and pulse widths of 5 ns. Two laser setups are used for VisPD. In order to measure the dependence on pulse energy, the Horizon is used. The laser is focused into the high pressure ion trap using a 1000 mm focal length convergent lens. A mechanical shutter, synchronized with the mass spectrometer, is used to stop the beam at all times except the “ion activation window,” that is, the time after ion accumulation and before mass analysis. A single laser pulse is used for the irradiation of the trapped ions, and the normalized collision energy is kept at zero during irradiation. The laser pulse energy is adjusted using a half waveplate and polarizing beam splitter, and is measured using a power meter (Ophir). At each laser power, chromophore-tagged peptides were trapped and irradiated sequentially and the photodissociation mass spectrum averaged over a period of 2 min. The branching ratios and fragmentation yields are calculated as explained above.

For measurement of the collisional cooling time, both Horizon and PantherEx are used. Both lasers are set to 545 nm, and combined using a Brewster window. The pulse energy of the PantherEx is controlled using a half waveplate and the Brewster window. The pulse energy of both lasers is set such that the fragmentation onset is just reached. The delay between the laser pulses is controlled by a delay box (SRS DG645) and monitored using a photodiode. Lasers are focused into either the high or low pressure trap using a 1000 mm focal length convergent lens, and a single pulse of each laser is used as described above.

## Results

### Fragmentation Yield as a Function of Pulse Energy

**Figure 1:**
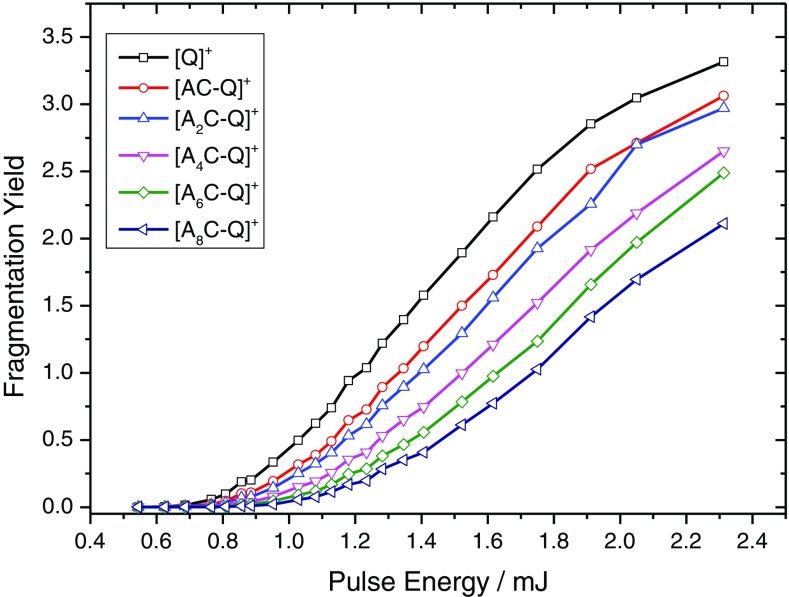
Fragmentation yield as a function of the laser pulse energy for mass selected [QSY7]^+^ (black squares), [AC-Q] ^+^ (red circles), [A_2_C-Q] ^+^ (blue triangles up), [A_4_C-Q] ^+^ (pink triangles down), [A_6_C-Q] ^+^ (green diamonds) and [A_8_C-Q] ^+^ (navy triangles left)

The dependence of the photofragmentation yield of QSY7-tagged peptides A_n_C as a function of the laser pulse energy is shown in Figure 1 at 545 nm. In each profile, there is a non-zero onset followed by a quasi-linear rise and, in the case of the smaller systems, a plateau signaling the onset of saturation. As the size of the peptide is increased, the value of the onset of photofragmentation is increased and the slope of the linear increase with pulse energy is decreased. The onset of each curve may be determined by a linear extrapolation of the linearly increasing portion of the curve to the baseline, and the gradient may also be extracted from the same data (see Figure S2 and Table S1). It can be noted that the photofragmentation profiles as a function of the pulse energy obtained resemble those obtained when performing IRMPD measurements as a function of the activation time [30]. It is therefore sensible to compare the onset and slope to the number of vibrational degrees of freedom for each system, Figure S3. There is a linear correlation between both the onset and slope with the number of vibrational degrees of freedom.

### Collisional Cooling of Trapped Ions Is Observed

**Figure 2:**
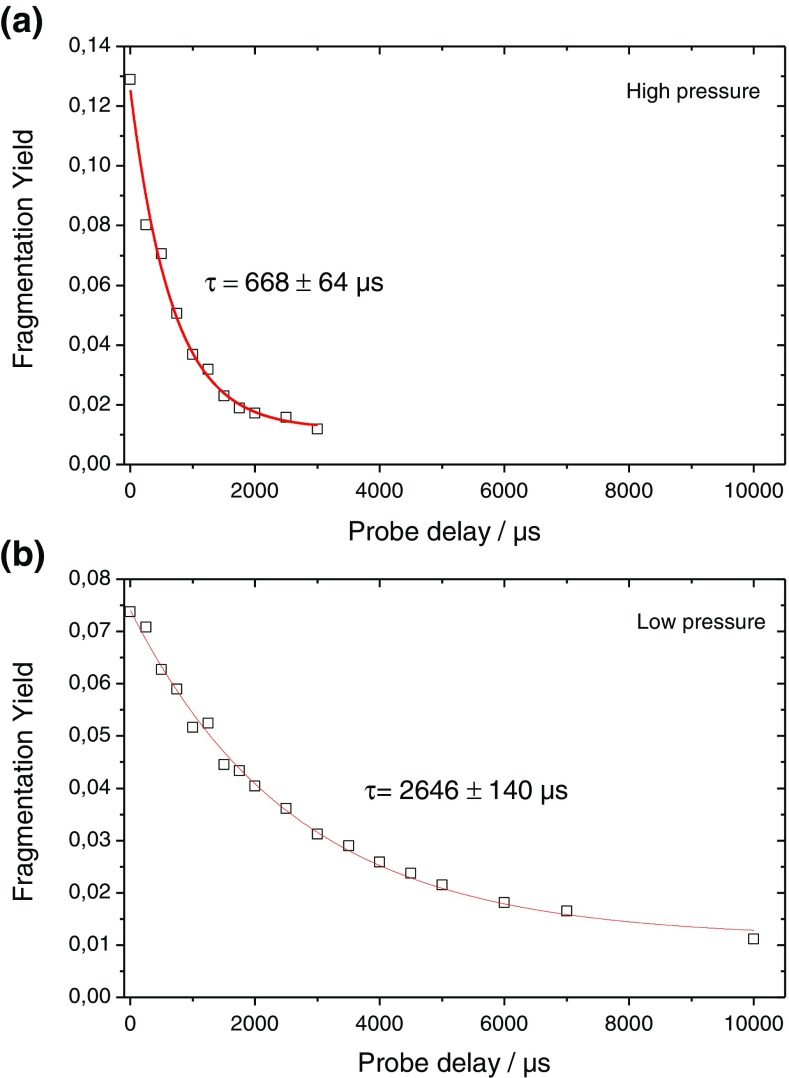
Pump + probe fragmentation yield as a function of the probe delay for mass selected [A_2_C-Q]^+^ at 545 nm taken in either the high pressure (**a**) or low pressure (**b**) cells of the LTQ Velos

To show there is an increase in the temperature of the trapped ions upon irradiation with visible photons, two lasers with pulse energies fixed to be just below the observed photofragmentation onset were used with a well-defined delay between each pulse, as described above. Figure S4 shows the mass spectrum of mass selected [A_2_C-Q]^+^ following irradiation with 545 nm photons by pump laser only (middle), probe laser only (bottom), and both lasers with a 30 ns delay (top). Very small levels of fragmentation are observed when using either pump or probe laser alone (a small amount of fragmentation is required to assure good alignment of both lasers). A large enhancement in the photofragmentation yield is observed when the trapped ions are irradiated with both laser beams simultaneously. Notice that the 30 ns delay ensures there is no temporal overlap of the pump and probe lasers. The fragmentation can be quantified as described in the Methods Section above. Figure 2 shows the photofragmentation yield as a function of the delay between pump and probe laser in the high pressure (Figure 2a) and low pressure (Figure 2b) cells. Both curves exhibit a mono exponential decay with a time constant of 668 ± 64 μs and 2646 ± 140 μs, respectively. This large increase in the relaxation timescale when moving to lower pressure is expected if collisional cooling is the dominant relaxation mechanism to reduce the internal temperature following irradiation with the pump laser. Thus, it is possible to conclude that photon absorption by QSY7 during the first laser pulse leads to heating of the system to which it is tagged, and that the system relaxes by collisional cooling.

### Branching Ratios Observed Following CID and VisPD

**Figure 3:**
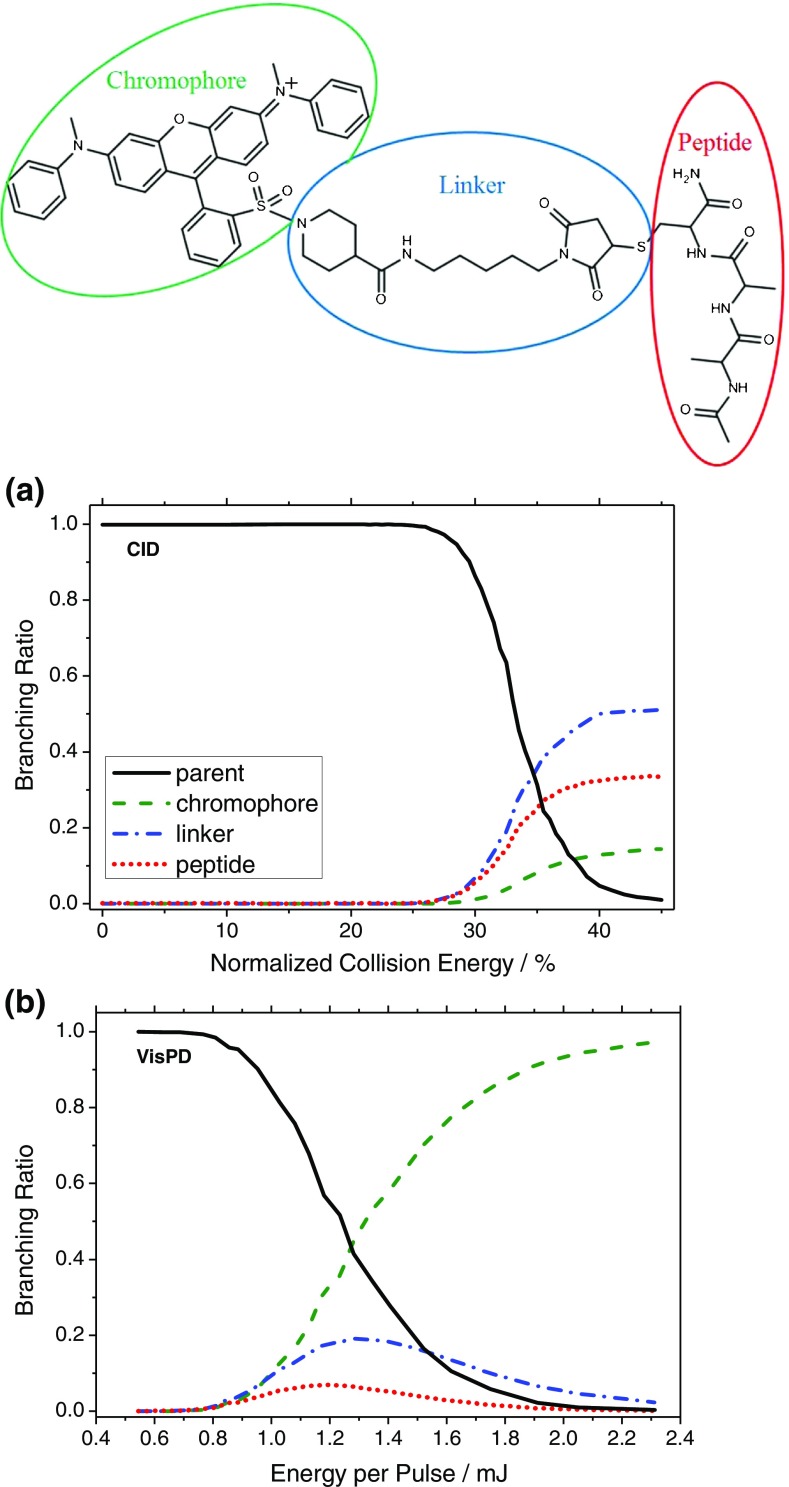
Schematic (top) of how the fragmentation channels can be divided into chromophore based (green), linker based (blue), or peptide based (red), shown here for [A_2_C-Q]^+^. Branching ratio for the parent (black continuous), chromophore (green dashed), linker (blue dot-dashed), and peptide (red dotted)

As it has been demonstrated that irradiation by visible photons causes the QSY7-tagged peptides to be heated, it is instructive to compare the fragmentation either as a function of the collisional energy in CID, or as a function of the pulse energy in VisPD experiments. To do this, the molecule was partitioned into three regions: the chromophore, linker, and peptide regions, Figure 3 (top panel), which is possible since there is only a single, fixed charge located at a defined point on the chromophore (see Figures S5 and S6, and Table S2 for an example mass spectrum and a detailed assignment of the observed fragmentation). The branching ratio of fragments for each region are determined as described above for different values of the pulse energy or collision voltage for [A_4_C-Q]^+^, considering first the branching ratios for CID, Figure 3a. Above 25% normalized collision energy (NCE) fragments associated with breaking of the linker chain and of the peptide are observed. The linker fragments are dominated by cleavage of the C_β_–S bond of the cysteine side chain, as previously observed for maleimide-tagged peptide-chromophore species [24, 25]. The peptide fragments are dominated by *b/y* ions with small neutral losses also observed. Above 30% NCE fragmentation related to the chromophore is also observed with a small (<20%) branching ratio. The chromophore-based fragments are identical to those previously reported for QSY7 [23]. The sigmoidal shape of the survival ratio has been previously observed for various species in CID experiments [31].

Contrastingly, the branching ratios as a function of the pulse energy, Figure 3b, show that the chromophore-based fragmentation channels are dominant. It is also interesting to note that in contrast to CID, fragmentation in all three regions is opened at commensurate pulse energies. The fragmentation channels observed are identical to those observed in CID, with only branching ratios differing. The branching ratio for linker and peptide fragments are peaked around 1.3 mJ/pulse and 1.2 mJ/pulse, respectively. This is because both linker and peptide fragments contain an intact chromophore, which is capable of undergoing secondary absorption and fragmentation events. Indeed, at very high pulse energies almost all fragments observed belong to the chromophore region attributable to high rates of secondary fragmentation and the charge being fixed on the chromophore. However, at pulse energies between 0.8 and 1.2 mJ/pulse, chromophore and linker fragmentation is dominant with peptide fragmentation being a minor (<10%) channel. This suggests that although fragmentation in CID and VisPD both occur via heating in these systems, the mechanism causing this is different between the two methods.

### Simulation of the Number of Photons Absorbed to Cause Photodissociation

It is possible to write a general equation for the processes that may occur when considering the unimolecular decay of an excited ion, Equation 1, as:

1 $$ {\left[ AB\right]}^{+}\rightleftharpoons {\left[ AB\right]}^{+*}\to {\left[A\right]}^{+}+\left[B\right] $$

In the VisPD experiments, where the collision energy is kept to zero, the rate of excitation is governed entirely by the rate of photon absorption. The deactivation of this excited state may either take place dissociatively, via photon emission, or via collisional cooling. Here, the chromophore is known to be a fluorescence quencher and hence photon emission is not an accessible relaxation channel. Furthermore, it was shown above that the collisional cooling rate is of the order of 1 ms, which is six orders of magnitude longer than the excitation process. If we consider a simple model where, for a given system, *n* photons must be absorbed within the 5 ns laser pulse in order to reach the dissociation limit. Given that the collisional cooling rate is slow compared with the excitation time, it is possible to assume that all of the systems that dissociate have reached the dissociation limit and have undergone dissociation before collisional cooling, i.e., that the rate of dissociation is much faster than the collisional cooling rate. This may not be valid at higher pressure. Note that the dissociation limit includes a kinetic shift so that the onset of observable dissociation for large molecules can significantly exceed the energy threshold for which the dissociation becomes thermodynamically possible [11, 32, 33].

Since the excited state life-time of similar rhodamine derivatives is in the range of 10 ps and an excited state lifetime in the lower ps range has been assigned to QSY7 by excited to excited state transition considerations when using different excitation laser pulse widths, it can be considered that sequential absorption of many photons by the chromophore within the laser pulse duration are independent events [13, 34, 35]. Thus, they may then be described by Poisson statistics. It is possible to construct a simple model to reproduce the shape of the parent survival curve presented in Figure 3b by considering the probability that for a given pulse energy, a molecule will absorb less than *n* photons and then survive laser irradiation. In this model, we assume that after absorption of *n*-1 photons, the molecule will survive whilst it will dissociate after absorption of *n* photons. If *P(N,i)* is the probability that a system will absorb *i* photons, where *N* is the average number of photons absorbed during a single laser pulse, we can define the following sum as the probability that a parent molecule survives when irradiated at a given pulse energy, Equation 2, as:

2 $$ S(N) = {e}^{-N}{\displaystyle {\sum}_{i=0}^n\frac{N^i}{i!}} $$

**Figure 4:**
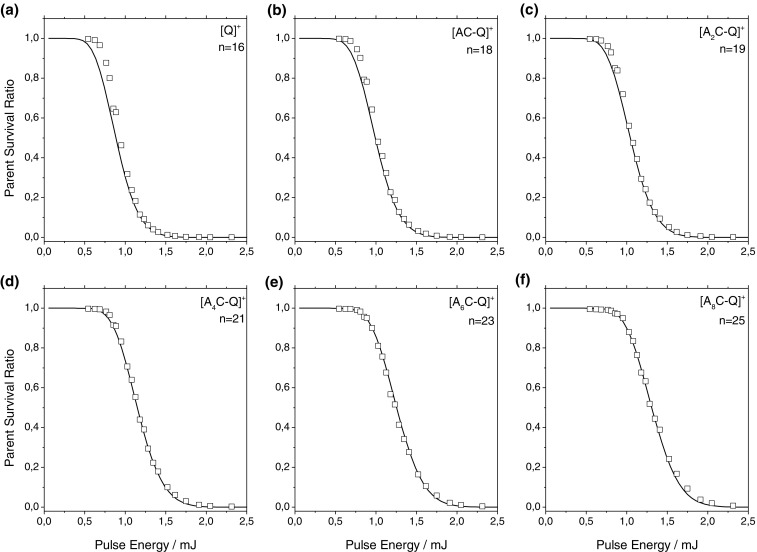
Experimental (squares) breakdown curves as a function of pulse energy for mass selected (**a**) [Q]^+^, (**b**) [AC-Q] ^+^, (**c**) [A_2_C-Q] ^+^, (**d**) [A_4_C-Q] ^+^, (**e**) [A_6_C-Q] ^+^, and (**f**) [A_8_C-Q] ^+^. Optimal simulated curves (solid lines) were calculated for n photons absorbed

The average number of photons absorbed by a single molecule irradiated by a laser pulse is proportional to the absorption cross-section of the chromophore *σ* and the photon flux of the laser pulse, i.e., Equation 3,

3 $$ N=\frac{\sigma \lambda {E}_p}{hcA} $$

where *λ* = 545nm is the wavelength, *E*_*p*_ is the laser pulse energy, and *A* the cross-sectional area of the laser at the interaction point with the ions. The absorption cross-section of QSY7 is given by the supplier as 3.66 × 10^–16^ cm^2^, and the laser profile in the ion trap is measured to be approximately circular with a diameter of ~2.5 mm, giving a cross-sectional area of ~0.05 cm^2^. The simulated curves can be found in Figure 4, with the corresponding values of *n* found in Table 1. The simulations very well reproduce the survival curves, especially for the larger system, which indicates that the model of sequential photon absorption is valid. The values for the number of photons required to induce photofragmentation vary from 16 to 25. The largest error in the simulation comes from the estimation of beam size, its overlap with the ion cloud, and the use of a simple stepwise model for dissociation. It is possible that the covalent bonding to the peptide has some effect on the absorption properties, as has been observed in several maleimide dyes, which can explain the disagreement for the smaller systems [28]. Moreover, for the isolated chromophore, the weakest bonds are no longer present and dissociation energies are thus expected to be slightly higher for this system. Alternatively, it may be the case that the nature of the IVR process is different where the peptide and linker are not present.

**Table 1:** Vibrational Degrees of Freedom and Number of Photons Absorbed as Determined by Fitting Experimental Data and Simulation

Species	Vibrational degrees of freedom	Number photons required for photofragmentation
[Q]^+^	318	16
[AC-Q]^+^	408	18
[A_2_C-Q]^+^	438	19
[A_4_C-Q]^+^	498	21
[A_6_C-Q]^+^	564	23
[A_8_C-Q]^+^	618	25

It is important to note that there is a linear dependence of the number of photons required for fragmentation (and hence the total energy deposited into the system) and the number of vibrational degrees each system possesses (see Figure S7). This linear dependence of activation energy required against excitation has been previously reported for polyethylene glycols in CID experiments [31].

## Discussion

In order to disentangle the photofragmentation of the chromophore-tagged peptide systems, it is instructive to first summarize the conclusions drawn from the results. The observation of a sigmoidal profile for photofragmentation yield as a function of the pulse energy, a fragmentation onset that depends on the size of the tagged peptide, and the observation of collisional cooling in pump-probe experiments show that absorption of a 545 nm photon by the chromophore leads to a heating of the system and that multiple absorption events are required for its subsequent fragmentation. Furthermore, it has been shown with a simple model that it takes between 16 and 25 photons to induce photofragmentation depending on the size of the system. Comparison of CID and VisPD, however, shows different fragmentation behavior, and it must be concluded that the process behind the fragmentation is not the same. The heating that occurs during the CID process is isotropic, and thus it is expected that fragmentation channels with the lowest activation energy will be accessed first, regardless of their location in the system.

To further understand the fragmentation following excitation with multiple visible photons, greater consideration must be given to the radiationless relaxation of the chromophore. Previous work on a dye closely related to QSY7 has shown that this fluorescence quenching is due to ultrafast IC related to rotational motion of the bulky side chains, and found that the IC occurs in less than 10 ps [34, 36, 37]. In QSY7, therefore, it is possible to invoke a similar IC process based upon low energy torsional modes of the methylamino-benzene moieties. This conclusion is supported by comparison of the calculated S_0_ and S_1_ state minimum energy structures (time-dependent density functional theory calculations, Figure S8), where the largest change in conformation is related to these torsional modes. Since IC is usually associated with a small number of vibrational degrees of freedom that couple the ground and excited states, this means that upon IC a small number of chromophore-based ground state normal modes will be very highly vibrationally excited.

Following IC, it is expected that IVR will take place and distribute the energy throughout the molecule. It has been demonstrated above that the parent survival curve can be well simulated, and that between 16 and 25 photons are required to induce fragmentation depending upon the size of the system. The number of photons necessary to absorb in order to induce dissociation displays a linear dependence on the number of vibrational normal modes, which is consistent with the IVR step before fragmentation, Figure S7. This is consistent with prior observations in small PAHs [38]. Furthermore, the observation of fragmentation channels associated with the linker and peptide show that part of the energy must be redistributed across the entire system despite excitation initially localized at the chromophore, which points to a global heating of the whole system prior to fragmentation.

**Scheme 1:**
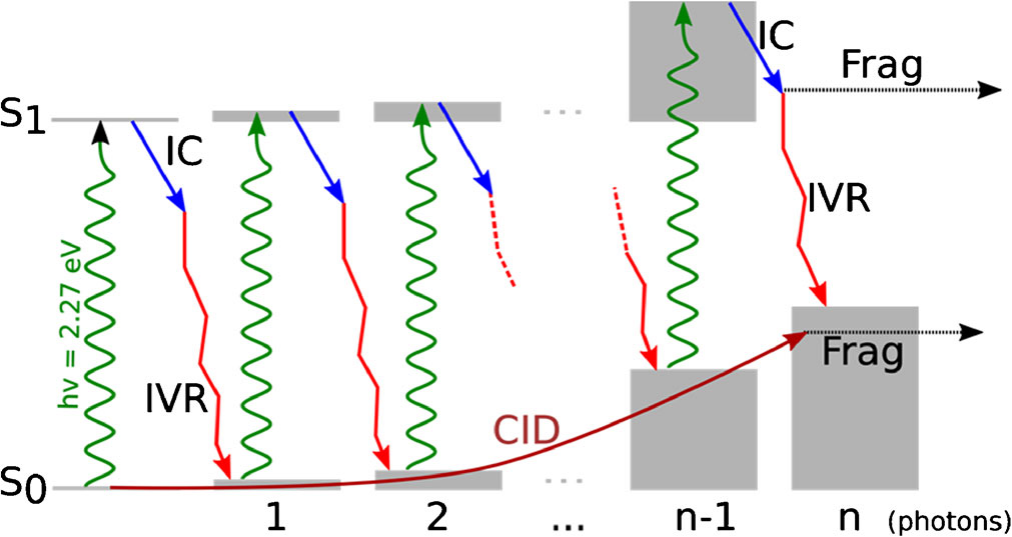
Schematics of the CID and VisPD processes leading to fragmentation in chromophore-tagged peptides. In VisPD, each 545 nm photon absorbed brings 2.27 eV (green arrows up) to the system, which then undergoes IC (blue arrows down) followed by IVR (broken red arrows down) that slowly heat up the system (grey bars). When the n^th^ photon is absorbed, direct fragmentation is competing with IVR, leading to possibly high energy fragments. Conversely, collisions (CID arrow up) heat up the system until it reaches the dissociation limit, which includes a kinetic shift

It is now possible to understand the difference in fragmentation following CID or VisPD. Consider a system that must absorb *n* photons in order to induce dissociation. The sequential absorption of the *n*-1 photons followed by IC and IVR lead to the heating of the whole system, see Scheme 1 (left panels). At this point, absorption of one more photon will induce photofragmentation. Upon absorption of this final photon, the system will have a highly non-isotropic energy distribution immediately following IC; all the excess energy of the final photon will be localized in vibrational normal modes on the chromophore. This highly anisotropic energy distribution means that chromophore-based fragmentation channels will be energetically accessible before fragmentation channels at locations distant from the chromophore, regardless of whether these channels open at lower energy (as indicated in the CID measurements), since IVR takes some time to redistribute energy. In particular, the C–S–N bonds represent a potential IVR bottleneck that could contribute to the different branching ratios.

## Conclusion

The photofragmentation properties of chromophore-tagged peptides differing in the number of alanine residues have been presented. The observation of a linear correlation between the size of the tagged peptide and the laser pulse energy required for photofragmentation onset, combined with the observation of collisional cooling, showed that photofragmentation occurs via sequential multiphoton absorption in a process similar to IRMPD. It was possible to simulate the parent survival curves for each of the tagged peptides studied in order to determine how many photons were required to induce fragmentation. Furthermore, it was shown that according to this simple model, the number of photons was linearly dependent on the number of vibrational normal modes, confirming that heating by visible light occurs. Comparison of CID and VisPD branching ratios of different fragmentation channels as a function of the energy showed that whilst CID shows predominantly peptide or linker fragmentation, VisPD is dominated by fragmentation of the chromophore. This difference can be understood as resulting from the isotropic heating of the system in CID with the highly anisotropic heating in VisPD caused by the absorption of the final photon. This is due to the IC populating a small number of normal modes with a large amount of energy, and fragmentation from these modes is faster than IVR. Thus, despite the sequential multiphoton absorption and heating of the system, chromophore-specific fragmentation is observed.

## Electronic supplementary material

Below is the link to the electronic supplementary material.

ESM 1 (PDF 695 kb)
