# Supplementary material for: Visible Multiphoton Dissociation of Chromophore-Tagged Peptides
Source: J Am Soc Mass Spectrom. 2017 Jul 28;28(10):2181–8. doi: 10.1007/s13361-017-1733-9 (PMC5594054; doi:10.1007/s13361-017-1733-9)
Supplement: Supplementary file 1 — (PDF 695 kb) [file 13361_2017_1733_MOESM1_ESM.pdf]

<u>**Supporting information**</u>

Visible Multiphoton Dissociation of Chromophore Tagged Peptides.

Mathilde Bouakil[a]*, Alexander Kulesza*[a]*, Steven Daly*[a]*, Luke MacAleese*[a]*, Rodolphe Antoine*[a]*, Philippe Dugourd**[a]

[a]Univ Lyon, Université Claude Bernard Lyon 1, CNRS, Institut Lumière Matière, F-69622, LYON, France

Corresponding Author

* Philippe.dugourd@univ-lyon1.fr

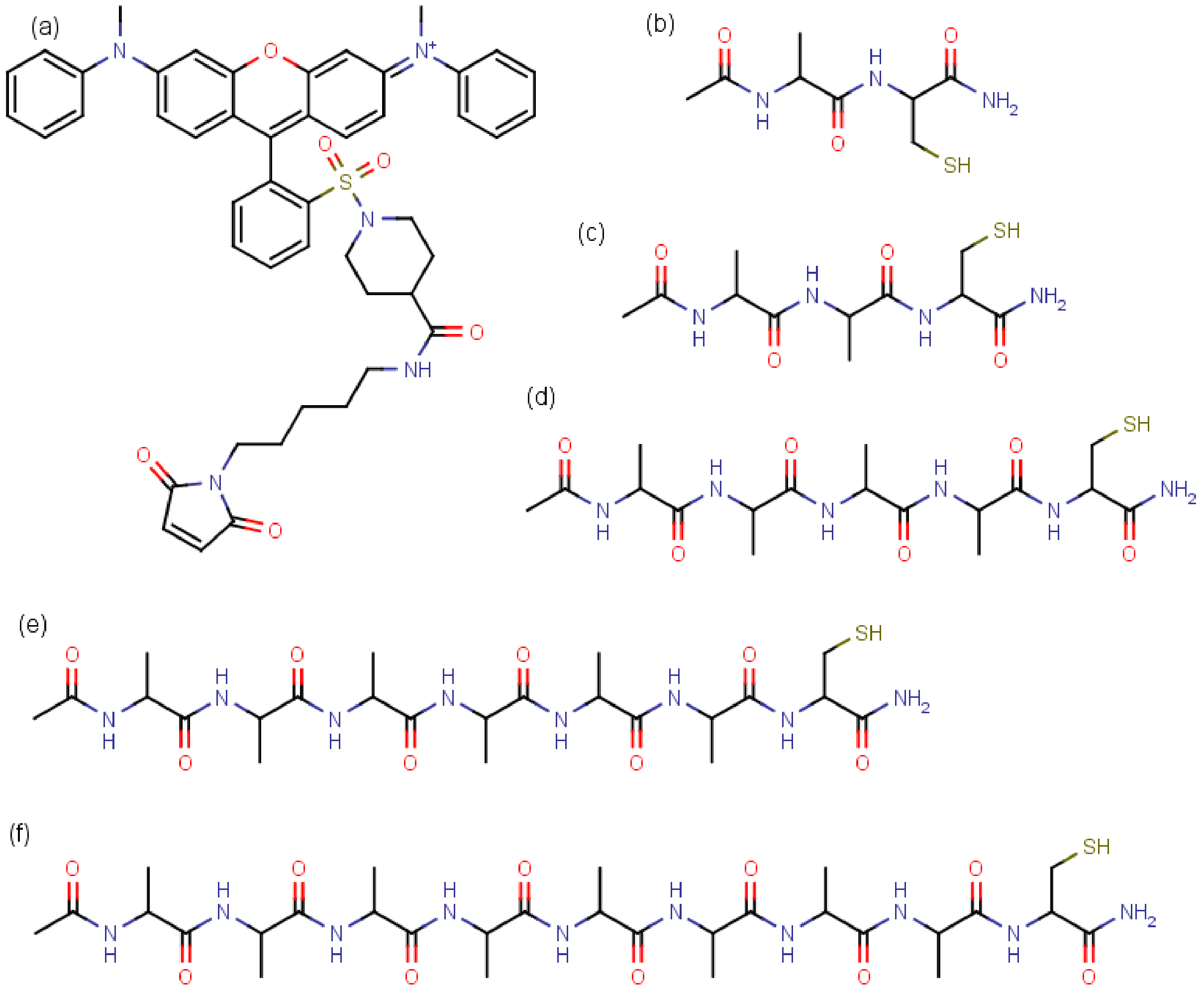

Figure S1. Structure of (a) QSY 7 C5 maleimide, (b) ace-AC-NH2, (c) ace-A_2C-NH2, (d) ace-A_4C-NH2, (e) ace-A_6C-NH2 and (f) ace-A_8C-NH2. The chromophore is covalently attached to the thiol moiety of the cysteine side chain to form a thio-ether.

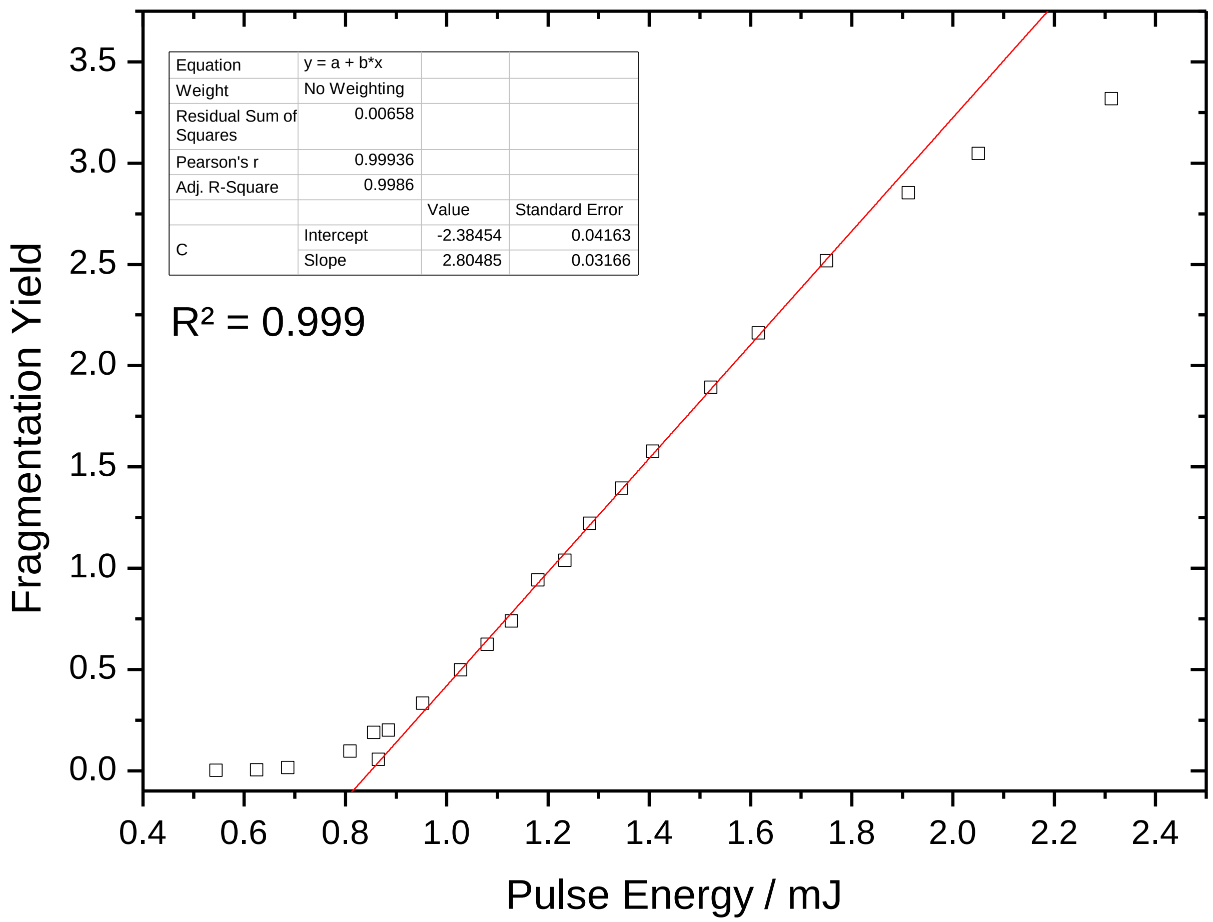

Figure S2. Example of straight line extrapolation of fragmentation yield as a function of the pulse energy, here shown for $[QSY7]^{+}$.

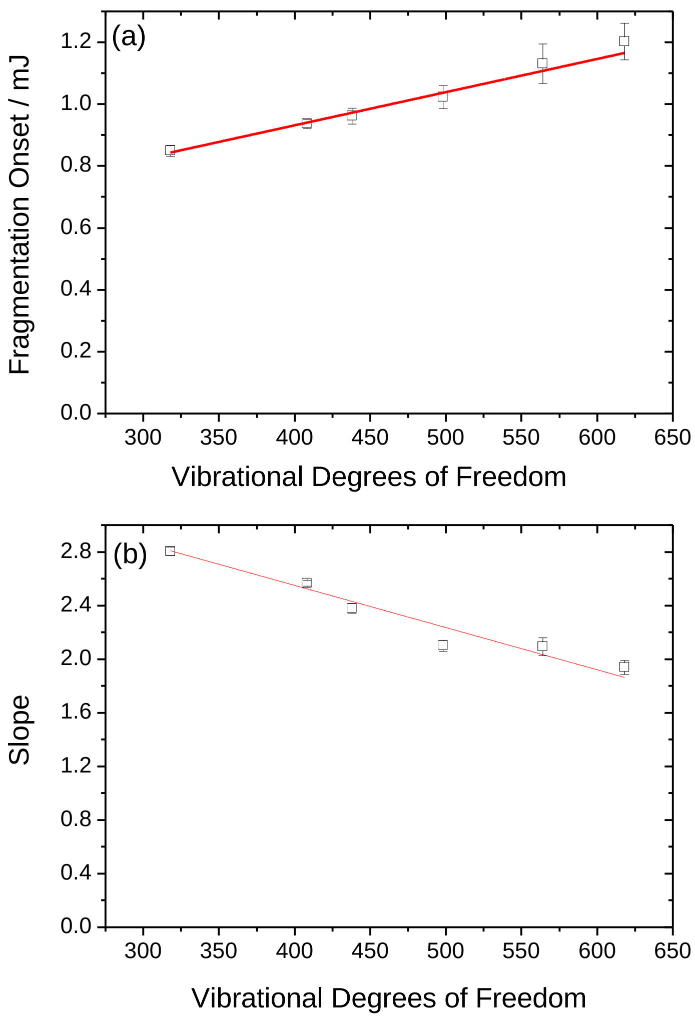

Figure S3. Fragmentation onset (a) and slope (b) of the linear fit of the photofragmentation yield versus laser pulse energy plots in Figure 1 as a function of the number of vibrational degrees of freedom.

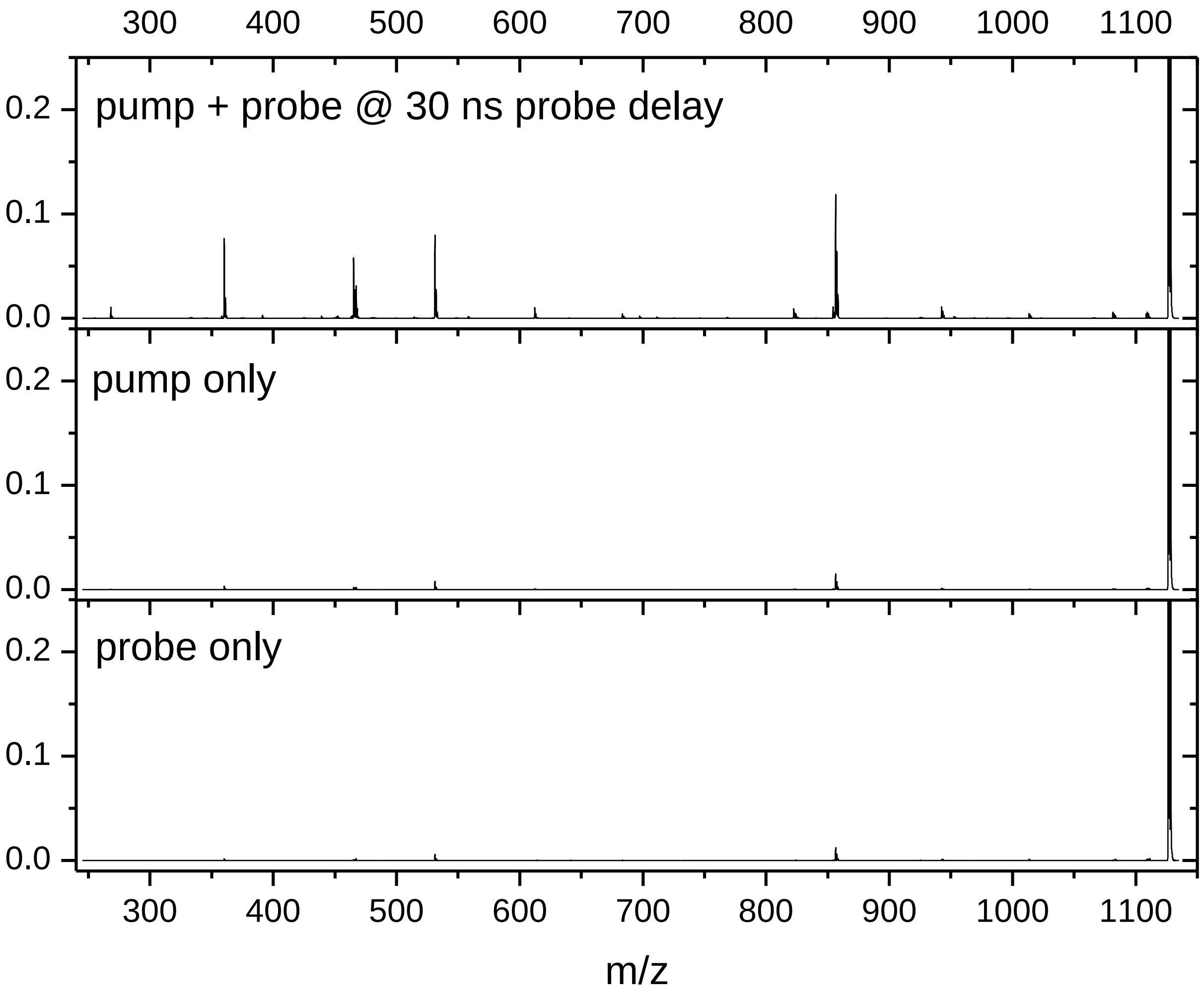

Figure S4. LID mass spectrum of mass selected $[A_4C\text{-}Q]^+$ following irradiation with 545 nm photons. The top panel shows both pump and probe lasers active with a probe delay of 30 ns. The middle and bottom panels show the spectrum for pump and probe lasers only. Intensities are normalized onto the parent peak.

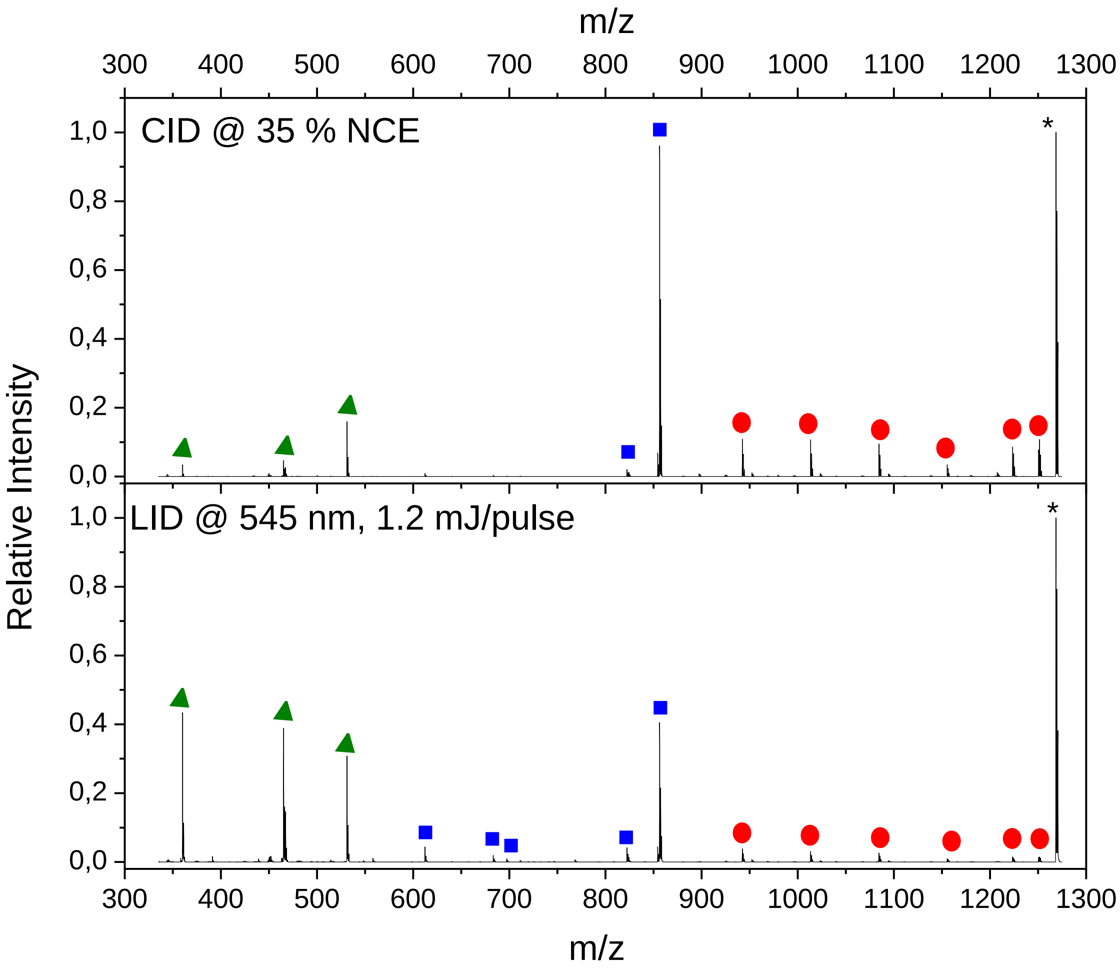

Figure S5. Mass spectra of mass selected $[A_4C\text{-}Q]^+$ following either CID with normalized collison energy of 35 % (top) or LID at 545 nm with a laser pulse energy of 1.2 mJ. The green triangles, blue squares and red circles represent fragments associated with the chromophore, linker, and peptide respectively. The black asterisk denotes the parent.

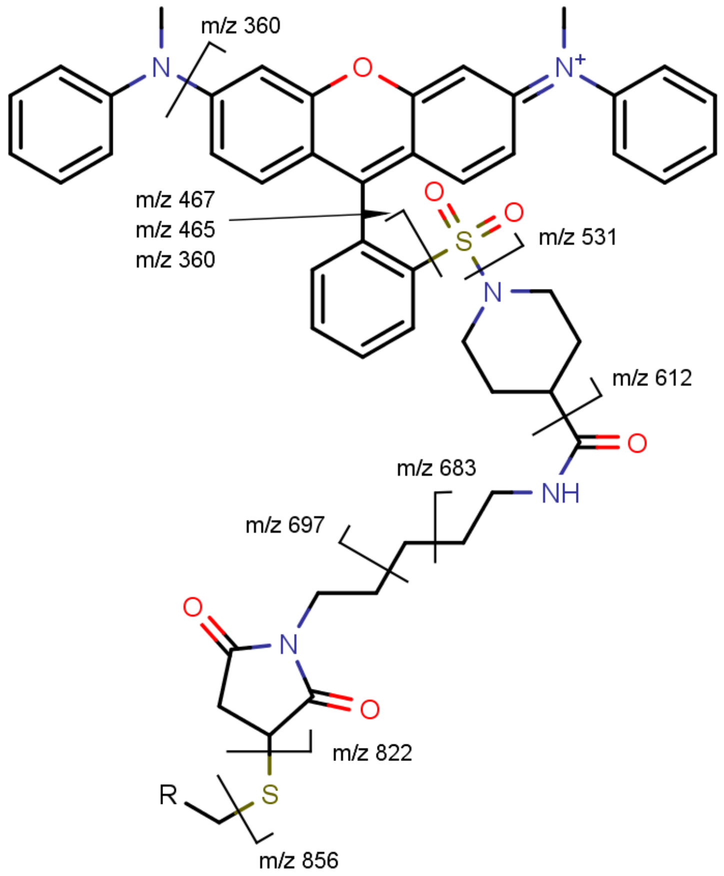

Figure S6. Schematic diagram showing the origin of the different fragmentation channels noted in Table S2, which are specific to the linker or the chromophore (peptide fragments are denoted as usual in Table S2). Note that the C – S bond is broken for m/z 467, 465 and 360 Da, denotes by the arrow. For m/z 360 Da, the C-N bond between xanthene and n-methylamino benzene is also broken. The R denotes the position of the C_α of the cysteine residue, and hence where the remainder of the peptide can be found.

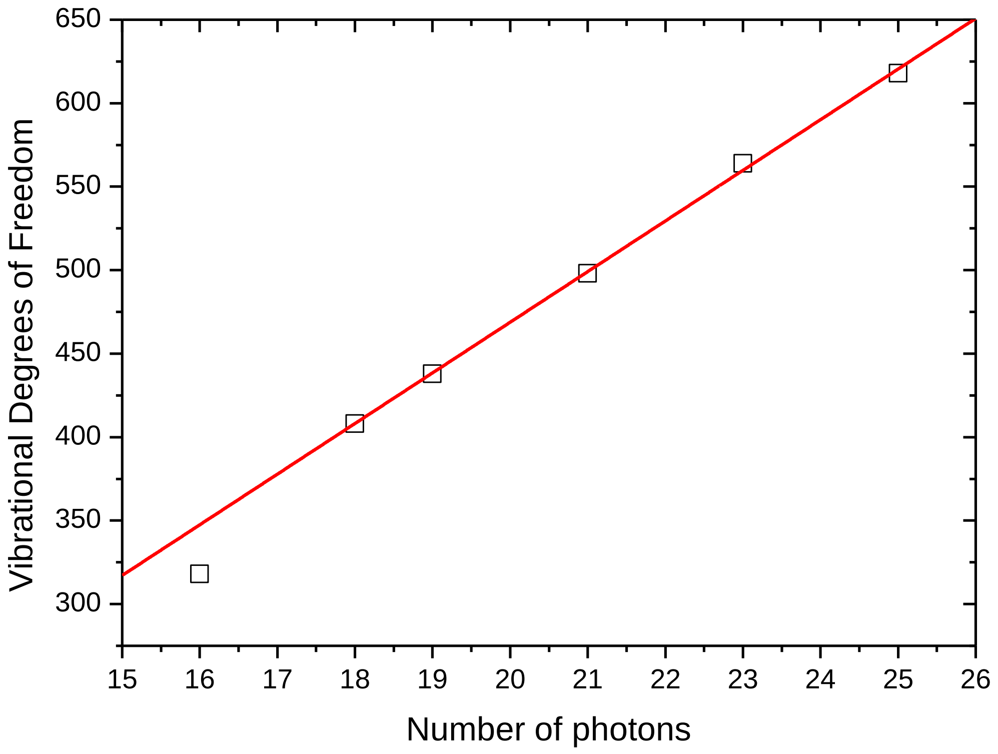

Figure S7. Number of vibrational degrees of freedom for chromophore tagged peptides as a function of the number of photons absorbed as determined by the simulation of the parent survival ratio.

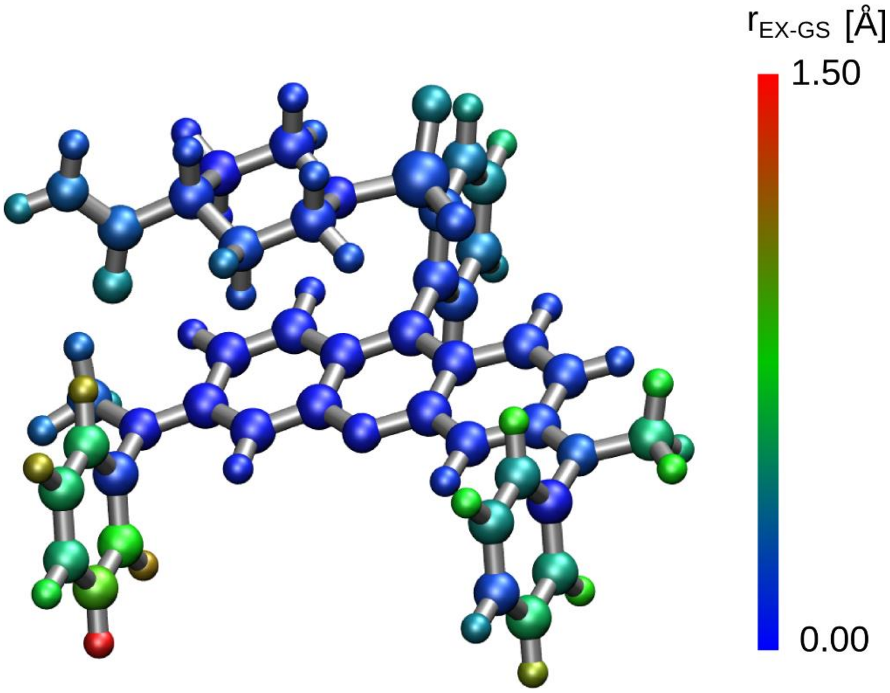

Figure S8. Representation of the geometry change between S0 and S1 minimum energy structures for QSY7 calculated at the ωB97X-D[1]/def2SVP[2] level using Gaussian09 Rev D.01[3]. Color coding (see legend on the right) of the atoms is done using the length of each atom's displacement vector between ground state (GS) and relaxed excited state (EX) geometry. It is seen that torsional motion of the benzene moieties is the largest change.

1. Chai, J.-D. & Head-Gordon, M. Long-range corrected hybrid density functionals with damped atom-atom dispersion corrections. *Phys. Chem. Chem. Phys.* **10,** 6615–6620 (2008).

2. Weigend, F. & Ahlrichs, R. Balanced basis sets of split valence, triple zeta valence and quadruple zeta valence quality for H to Rn: Design and assessment of accuracy. *Phys. Chem. Chem. Phys.* **7,** 3297–305 (2005).

3. Frisch, M. J. *et al.* Gaussian 09, Revision D.01. (2009).

Species	Onset / mJ	slope
$[QSY7]^+$	0.85 ± 0.02	2.80 ±0.03
$[AC\text{-}Q]^+$	0.94 ± 0.01	2.57 ± 0.02
$[A_2C\text{-}Q]^+$	0.96 ± 0.03	2.38 ± 0.04
$[A_4C\text{-}Q]^+$	1.02 ± 0.04	2.10 ± 0.04
$[A_6C\text{-}Q]^+$	1.13 ± 0.06	2.10 ± 0.07
$[A_8C\text{-}Q]^+$	1.20 ± 0.06	1.94 ± 0.05

Table S1. Fragmentation onset and slope from linear fitting of the straight line region of the plot of photofragmentation yield versus laser pulse energy, see Figure S2.

m/z	Loss	Relative Intensity		Assignment
		CID	LID	
1268.6	0	1	1	parent
1251.6	17	0.11	0.015	NH_3 loss
1250.6	18	0.077	0.013	H_2O loss
1223.52	45.08	0.086	0.015	NH_2-CH=O (C-term)
1207.52	61.08	0.013	0.0022	NH_3 + CH_3CHO?
1155.52	113.08	0.034	0.0096	y4
1084.48	184.12	0.095	0.027	y3
1013.44	255.16	0.11	0.032	y2
952.4	316.2	0.01	0.0074	a4 - H2O
942.4	326.2	0.11	0.039	y1
856.36	412.24	0.96	0.4	C - S break side chain, H on S
854.36	414.24	0.068	0.044	C - S break side chain, H on C
822.4	446.2	0.02	0.042	Reverse grafting reaction
697.35	571.25	0.0011	0.01	C_5 linker C-C
683.34	585.26	0.0036	0.02	C_5 linker C-C
612.26	656.34	0.0086	0.044	Piperidine - C break
531.2	737.4	0.16	0.31	N - S break
467.24	801.36	0.026	0.15	C - S break chromophore
465.24	803.36	0.046	0.39	C - S break chromophore
360.2	908.4	0.034	0.43	C-S break + side chain loss

Table S2. m/z ratio, neutral loss, relative intensity in the CID and LID spectrum and assignment for the CID and LID mass spectra shown in Figure S3. Note that only fragmentation channels with > 1 % relative intensity in one of the two spectra have been assigned for clarity
